# Chest X-ray: an examination that has been in use for centuries but is still essential, especially in the clinical management of newborns in the neonatal intensive care unit

**DOI:** 10.1590/0100-3984.2018.51.1e2

**Published:** 2018

**Authors:** Sara Reis Teixeira, Aline Naves

**Affiliations:** 1 MD, PhD, Attending Physician at the Centro de Ciências das Imagens e Física Médica (CCIFM) of the Hospital das Clínicas da Faculdade de Medicina de Ribeirão Preto da Universidade de São Paulo (HCFMRP-USP), Ribeirão Preto, SP, Brazil. E-mail: steixeira@hcrp.usp.br; 2 MD, Physician at the Centro de Ciências das Imagens e Física Médica (CCIFM) of the Hospital das Clínicas da Faculdade de Medicina de Ribeirão Preto da Universidade de São Paulo (HCFMRP-USP), Ribeirão Preto, SP, Brazil

The most common causes of admission to a neonatal intensive care unit (NICU) are prematurity and low birth weight, which collectively account for up to 69% of hospitalizations in referral NICUs, including those in Brazil^1-4^. Respiratory complications, notably respiratory distress syndrome of prematurity, are major causes of mortality in the NICU^3,4^.

In the management of neonatal lung diseases that require NICU admission, chest radiography plays a fundamental role in the initial diagnosis of major clinical alterations of the respiratory profile and is the standard procedure to determine the positioning of probes, tubes, and catheters^5^. Despite the technological advances in diagnostic imaging, with the addition of various new modalities in recent decades, chest radiography continues to be the most widely used radiological modality in NICUs^6^.

Atelectasis is a common pulmonary alteration that can cause a sudden worsening of the clinical condition of a neonate, predisposing to infectious complications and increasing the need for ventilatory support^7^. Atelectasis is a sign of disease and, in isolation, is not suggestive of a specific diagnosis; hence, the need for clinical correlation. The treatment of atelectasis varies depending on its cause, duration, and severity^8,9^. The main mechanisms for the formation of atelectasis are airway obstruction, extrinsic compression, and increased surface tension between alveoli and bronchioles due to surfactant deficiency^9^. The main diseases that affect newborns with atelectasis include respiratory distress syndrome, meconium aspiration syndrome, pneumonia, pleural effusion, and pneumothorax^8^. Atelectasis can also be a consequence of inappropriate positioning of the endotracheal tube, which prevents adequate ventilation of the newborn^10^. Therefore, the detection of a malpositioned endotracheal tube should prompt its immediate correction^11^.

In this issue of Radiologia Brasileira, Alvares et al.^12^ discuss the role of chest X-ray in the evaluation of atelectasis in newborns with clinically treatable lung diseases, as well as its main forms of presentation, causal factors, and associated conditions, such as malposition of endotracheal tubes. The authors found that the endotracheal tube was positioned incorrectly in 87% of the patients and that malpositioning of the endotracheal tube was associated with prematurity and with a birth weight below 1000 g^12^. They also identified a trend towards an association between atelectasis in the upper lobe and malpositioning of the head. Their results corroborate those of other studies reporting that extremely-lowbirth-weight infants are more vulnerable to incorrectly placed tubes^11^. In addition, the malpositioning of the head of the neonate during conventional radiography is also a predisposing factor for inappropriate tracheal intubation, which can lead to atelectasis and adverse respiratory outcomes^11,13^. A distal positioning of the endotracheal tube occurs when the head is flexed, whereas proximal positioning occurs when the head is extended^13^.

Radiographs performed in the NICU result in increased exposure to ionizing radiation, not only for newborns, who are more sensitive to radiation than adults, but also for the NICU staff. Such radiographs also increase the risk of accidental removal of catheters or tubes and, consequently, intubation failure^14^. It is necessary to be aware of ionizing radiation dose reduction techniques and even to consider the use of other imaging modalities, such as ultrasonography^15^. To guarantee the quality of the examination and avoid additional exposure to ionizing radiation, with adequate attention paid to penetration, lung expansion, positioning of the neonate, and, especially, collimation is of great importance^16^. Although ultrasonography has been shown to be an alternative to conventional radiography for determining the position of the endotracheal tube, chest X-ray continues to be the gold-standard modality for this purpose^14,17^. Incorrect positioning of the newborn and malpositioning of the endotracheal tube can cause atelectasis. However, they are modifiable factors and can be directly controlled by the radiologist. With cautious, radiologists can contribute to improve outcomes in neonates.
